# Sample entropy reveals high discriminative power between young and elderly adults in short fMRI data sets

**DOI:** 10.3389/fninf.2014.00069

**Published:** 2014-07-23

**Authors:** Moses O. Sokunbi

**Affiliations:** ^1^MRC Centre for Neuropsychiatric Genetics and Genomics, Institute of Psychological Medicine and Clinical Neurosciences, Cardiff School of Medicine, Cardiff UniversityCardiff, UK; ^2^Imaging Science, Cardiff University Brain Research Imaging Centre, Cardiff UniversityCardiff, UK

**Keywords:** ageing, blood oxygen level dependent (BOLD), data length, functional magnetic resonance imaging (fMRI), noise level, sample entropy

## Abstract

Some studies have placed Sample entropy on the same data length constraint of 10^*m*^–20^*m*^ (*m*: pattern length) as approximate entropy, even though Sample entropy is largely independent of data length and displays relative consistency over a broader range of possible parameters (*r*, tolerance value; *m*, pattern length; *N*, data length) under circumstances where approximate entropy does not. This is particularly erroneous for some fMRI experiments where the working data length is less than 100 volumes (when *m* = 2). We therefore investigated whether Sample entropy is able to effectively discriminate fMRI data with data length, *N* less than 10^*m*^ (where *m* = 2) and *r* = 0.30, from a small group of 10 younger and 10 elderly adults, and the whole cohort of 43 younger and 43 elderly adults, that are significantly (*p* < 0.001) different in age. Ageing has been defined as a loss of entropy; where signal complexity decreases with age. For the small group analysis, the results of the whole brain analyses show that Sample entropy portrayed a good discriminatory ability for data lengths, 85 ≤ *N* ≤ 128, with an accuracy of 85% at *N* = 85 and 80% at *N* = 128, at *q* < 0.05. The regional analyses show that Sample entropy discriminated more brain regions at *N* = 128 than *N* = 85 and some regions common to both data lengths. As data length, *N* increased from 85 to 128, the noise level decreased. This was reflected in the accuracy of the whole brain analyses and the number of brain regions discriminated in the regional analyses. The whole brain analyses suggest that Sample entropy is relatively independent of data length, while the regional analyses show that fMRI data with length of 85 volumes is consistent with our hypothesis of a loss of entropy with ageing. In the whole cohort analysis, Sample entropy discriminated regionally between the younger and elderly adults only at *N* = 128. The whole cohort analysis at *N* = 85 was indicative of the ageing process but this indication was not significant (*p* > 0.05).

## Introduction

Recently, the application of entropy measures to investigate signal complexity and irregularity in human data has become quite popular (Yentes et al., 2013). Entropy values reflect the number of times the patterns in a signal are repeated and thus measure the randomness and predictability of stochastic process and in more general terms, increase with greater randomness (Sokunbi et al., 2013). The computation of entropy in biological data processing became a possible solution to the shortcomings posed by some metrics of nonlinear time series analysis techniques such as correlation dimension (Pritchard et al., 1994) and Lyapunov exponent (Wolf et al., 1985), which require a large data set (Eckmann and Ruelle, 1992) and assume that the time series is stationary (Grassberger and Procaccia, 1983), a feature normally not true for biological data. Approximate entropy (ApEn) (Pincus, 1991) and sample entropy (SampEn) (Richman and Moorman, 2000) are a few of the different types of entropy measures that have evolved from the concept of entropy. Regularity and complexity statistics such ApEn and SampEn are measures without the shortcomings that correlation dimension and Lyapunov exponent possess (Richman and Moorman, 2000). ApEn and SampEn can effectively discriminate both stochastic processes and noisy deterministic data sets in instances where measures such as spectral and autocorrelation analyses exhibit minimal distinctions (Pincus, 2001). They are also nearly unaffected by low level noise, are robust to occasional, very large or small artifacts and give meaningful information with a reasonable number of data points, and are finite for both stochastic and deterministic processes (Zhang and Roy, 2001).

The ApEn algorithm counts each sequence as matching itself to avoid the occurrence of ln(0) in the calculations, which led to the discussion of the bias of ApEn (Pincus, 1995). This bias causes ApEn to be heavily dependent on data length and uniformly lower than expected for short data lengths. Also, ApEn lacks relative consistency. To reduce this bias, SampEn was introduced as an improvement of ApEn where self-matches are excluded, i.e., vectors are not compared to themselves (Richman and Moorman, 2000). SampEn is the negative natural logarithm of the conditional probability that two sequences remain similar at the next point, where self-matches are not included in calculating the probability (Richman and Moorman, 2000). Hence, a lower value of SampEn also indicates more self-similarity in the time series. The algorithm of SampEn is simpler than the ApEn algorithm, requiring less time for computation. SampEn is largely independent of data length and displays relative consistency over a broader range of possible parameters (*r*, tolerance value; *m*, pattern length; *N*, data length) under circumstances where ApEn does not (Richman and Moorman, 2000).

SampEn has been used to characterize human data from a number of imaging modalities. To mention a few, it has been used to analyze the electroencephalogram (EEG) background activity in Alzheimer's disease patients (Abasolo et al., 2006). It has further been used to analyse the spontaneous magnetoencephalography (MEG) signals in patients with ADHD (Gomez et al., 2011) and to probe the complexity of resting state fMRI activity in adult patients with ADHD (Sokunbi et al., 2013). More recently, it has been used to examine the whole brain entropy patterns of a large cohort of normal subjects using fMRI (Wang et al., 2014). In all three brain imaging modalities, fMRI had the shortest data length. Since there are no laid down guidelines for choosing parameters to compute SampEn for all modalities of biomedical signals, some investigators have made suggestions for selecting parameters to use. Abasolo et al. (2006) suggested that to estimate SampEn of EEG accurately, a data length of 10^*m*^–20^*m*^ is required. Here, they used parameters *m* = 1, *r* = 0.25, and *N* = 1280 data length. In a recent study, Yentes et al. (2013) examined the robustness of ApEn and SampEn algorithms by exploring the effect of changing parameter values on short data sets using both theoretical and experimental data (musculoskeletal data with a data length of 200). In conclusion, they suggested to use a data length larger than 200, an *m* of 2, and to examine several *r*-values before selecting parameters. However, they also noted that SampEn was less sensitive to changes in data length and demonstrated fewer problems with relative consistency. Also, in another recent study of fMRI multiscale sample entropy analysis, SampEn was placed at the same data length threshold of 10^*m*^–20^*m*^ with ApEn (Yang et al., 2013), even though it is largely independent of data length and displays relative consistency under circumstances where ApEn does not (Richman and Moorman, 2000).

The developers of SampEn (Richman and Moorman, 2000) tested the consistency of SampEn for very short data sets using theoretical data (independent, identically distributed (i.i.d) Gaussian numbers) and found that SampEn statistics deviated from predictions for very short data sets. They calculated the biased results of SampEn (2, 0.2, *N*) for the range of 4≤ *N* ≤ 102. For Gaussian random numbers with *m* = 2 and *r* = 0.2, they found that the deviation was less than 3% for data lengths greater than 100 points but as high as 35% for data length of 15 points. They found that the bias of SampEn for very small data sets is largely due to “non-independence of templates” (Richman and Moorman, 2000) and that this bias appears to be present only for very small data lengths. They did not suggest or recommend a data length constraint for estimating SampEn.

FMRI is a potent research tool and has found more applications in research than clinical use. In contrast to EEG and MEG, fMRI possesses poor temporal resolution (in order of seconds) but excellent spatial specificity. As a result, most fMRI experiments are usually short, in the range of 100–200 data lengths. Prior data analysis, standard fMRI data processing requires that the first 3 or 4 volumes (data lengths) of fMRI data are discarded to enable signal conditioning. For fMRI data acquisitions of 100 data length, this results in a data length of 97 or 96. Our experience of characterizing fMRI data with SampEn shows that it is possible to obtain reliable results while using robust and optimal parameters such as *m* = 2, *r* = 0.46 (a high *r*-value) and a data length less than 100 (97 data points) (Sokunbi et al., 2013). We further tested the ability of SampEn to effectively discriminate fMRI data with data length, *N* less than10^*m*^ (where *m* = 2) using a resting state fMRI data set from a small group of 10 healthy right-handed younger and 10 right-handed elderly adults that are significantly (*p* < 0.001) different in age, extracted from the International Consortium for Brain Mapping (ICBM) resting state dataset. We also investigated the discriminatory ability of SampEn on the whole ICBM resting state cohort of 43 younger and 43 elderly adults that are significantly (*p* < 0.001) different in age. We used *m* = 2 which is superior to *m* = 1 since it allows more detailed reconstruction of the joint probabilistic dynamics of the time series (Pincus and Goldberger, 1994).

With normal ageing, there are declines in mental domains such as processing speed, reasoning, memory and executive functions, some of which is underpinned by a decline in a general cognitive factor (Deary et al., 2009). The bases for this decline are not fully understood. There has been progress in normal cognitive ageing from genetics, general health, biological processes, neurobiological changes, diet, lifestyle and many other areas of biomedical and psychosocial sciences. For example, the complexity of longitudinal physiological measurements such as EEG has been shown to vary with age and disease (Gaal et al., 2010). Complexity can be described as the difficulties associated with predicting a signal and this can be estimated by measuring the signal's entropy (Lu et al., 2008). Some studies have suggested that the characterization and analysis of the brain's output in terms of its complexity may reveal a better understanding of an individual's health and robustness (Goldberger et al., 2002), adaptive capacity in terms of brain ageing (Sokunbi et al., 2011) and diseases (Sokunbi et al., 2013, 2014), and *in-vivo* effect of drugs (Ferenets et al., 2007). Healthy systems portray chaotic and complex behaviors whereas pathological states exhibit predictable behaviors (Pool, 1989). Estimating the complexity of the blood oxygen level dependent (BOLD) fMRI signals can help to probe different aspects of complex signals brought about by ageing and disease, revealing subtle patterns which may provide fundamental insights that can lead to clinical and biomedical applications.

Investigators have argued that the pathway of change in the behavior and physiology of an organism with age and disease can either result in a decrease or an increase in the complexity of the system's output (Vaillancourt and Newell, 2002; Sokunbi et al., 2014). Vaillancourt and Newell (2002) postulate that the directional change in output complexity of a physiological or behavioral system with ageing or disease depends on the system having an underlying fixed point or an oscillatory attractor determining output. An attractor is the state to which a system returns to after perturbation (Vaillancourt and Newell, 2002). In the fixed-point attractor system, complexity decreases with age and disease (Sokunbi et al., 2013) while in the oscillatory attractor system complexity increases with age and disease (Sokunbi et al., 2014). Ageing has been defined as a loss of entropy (Lipsitz, 2004) and specific brain regions have been implicated in the ageing process (Craik and Salthouse, 2000). Also, functional entropy has been shown to increase with age (Yao et al., 2013). In the present analysis, we expect SampEn to decrease with age according to Lipsitz's (2004) entropy definition of ageing and Vaillancourt and Newell's (2002) fixed-point attractor postulate. Most importantly, we expect SampEn results at *N* less than 100 to be indicative of this ageing process since it is largely independent of data length and displays relative consistency (Richman and Moorman, 2000).

## Materials and methods

### Subjects

A small group of 10 healthy right-handed younger adults [5 male, mean age (22.40 ± 3.44)] and 10 healthy right-handed elderly adults [5 male, mean age (69.60 ± 9.25)] with significant (*p* < 0.001) age difference were extracted from the ICBM resting state dataset made publicly available in the 1000 Functional Connectomes project. The subjects used for the small group analysis are listed in the supplementary data, Table S1. The whole ICBM resting state cohort of 43 younger adults [21 male, mean age (29.05 ± 8.66)] and 43 elderly adults [20 male, mean age (59.33 ± 10.27)] with significant (*p* < 0.001) age difference was also investigated. The study was approved by the local research ethics committee and subjects had no history of neurological or psychiatric disorders. Written informed consent was obtained from the subjects. Information regarding this dataset is available at https://www.nitrc.org/projects/fcon_1000/.

### Brain imaging

Functional MR images were acquired with a T^*^_2_ weighted gradient echo echo-planar imaging sequence (EPI) using a standard head coil on a 3T scanner. A total of 23 axial slices were obtained for each of 133 volumes using a TR of 2 s and matrix 64 × 64. A total of 128 volumes of fMRI data remained after discarding the first five volumes to allow for signal conditioning. Subjects were asked to lie in the scanner with their eyes closed.

### Image pre-processing

FMRI data pre-processing were performed using SPM8 software (The Wellcome Department of Imaging Neuroscience, UCL, London, UK). The images were realigned to correct for head movement distortion. Temporal high pass filtering was performed (128 s) to reduce low frequency noise and spatial smoothing was performed to reduce white noise using the full-width at half maximum (FWHM) of the Gaussian smoothing kernel [8 8 8]. Each voxel time series was standardized to a mean of zero and standard deviation of unity. This allows a signal value of *r* (tolerance) to be used for all voxels independent of amplitude and variance.

### Computation of SampEn

The SampEn of a time series of length *N* (*x*_1_, *x*_2_,….., *x*_*N*_) can be computed from the given sets of equations (Sokunbi et al., 2013):

$$SampEn(m,r,N)=-\ln\left[\frac{U^{m\;+\;1}\left(r\right)}{U^{m}\left(r\right)}\right]$$

(1) $$U^{m}(r)={\left[N-m\tau \right]}^{-1}\sum_{i\;=\;1}^{N-m\tau }C_{i}^{m}(r)$$

Where

$$C_{i}^{m}\left(r\right)=\frac{B_{i}}{N-(m+1)\tau }$$

(2) $$B_{i}=\text{number of j where d}\left|X_{i},X_{j}\right|\leq r$$

(3) $$X_{i}\;=\left(x_{i},x_{i\;+\;\tau },\ldots ..,x_{i+(m\;-\;1)\tau }\right)$$

(4) $$X_{j}\;=\left(x_{j},x_{j\;+\;\tau },\ldots ..,x_{j+(m\;-\;1)\tau }\right)$$

1≤j≤N−mτ,j≠i

*N* specifies the data length, *m* is the pattern length, *r* is the tolerance value, and τ is the time delay as shown in Equation (1). In Equation (2), the two patterns *i* and *j* of *m* measurements of the time series are similar if the difference, d |*X*_*i*_, *X*_*j*_|, between any pair of corresponding measurements of *X*_*i*_ and *X*_*j*_ is less than, or equal to, *r*. In Equations (3 and 4), *X*_*i*_ and *X*_*j*_ are pattern vectors (length m) whose components are time-delayed versions of the elements in the original time series with time delay, τ.

**Figure 1:**
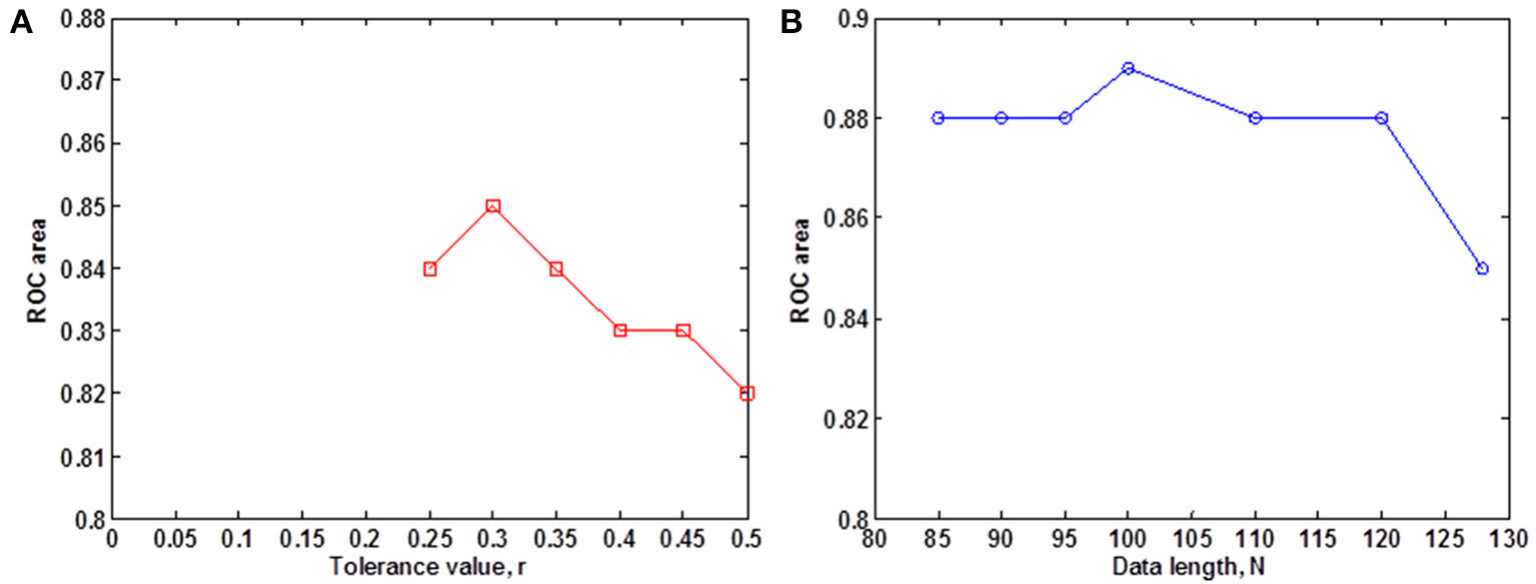
**Small group analysis. (A)** ROC area for determining the optimal *r*-value for fMRI data of 128 volumes, for 0.05 ≤ *r* ≤ 0.5 at intervals of 0.05. The optimal *r*-value was obtained at *r* = 0.30; **(B)** ROC area of SampEn (*m* = 2, *r* = 0.30, 85 ≤ *N* ≤ 128) for fMRI data lengths *N*. SampEn shows good discriminating ability and relative consistency for all the data lengths.

We evaluated the ability of SampEn to discriminate the younger from the elderly adults, using the value of the receiver operating characteristic (ROC) area (Zweig and Campbell, 1993). ROC areas are used as a guide to classify the precision of a diagnostic test. Areas with values between 0.90 and 1 indicate that the precision of the diagnostic test is excellent, when the values are between 0.80 and 0.89, it means the test is good. It is fair if the area values are between 0.70 and 0.79, poor when the area is between 0.60 and 0.69 and bad for values ranging from 0.50 to 0.59. Using the small group of 10 younger and 10 elderly adults, we determined the optimal *r*-value where this discrimination occurs by computing the ROC area for a range of *r*-values. The ROC area was computed from the mean whole brain SampEn values of each subject in the small group using a robust value of *m* = 2 (Pincus and Goldberger, 1994), data length *N* = 128 and by varying the *r*-value from 0.05 to 0.5 at intervals of 0.05. Figure 1A shows that this optimal *r*-value occurred at *r* = 0.30.

Whole brain SampEn was computed for each subject in the small group using *m* = 2, the optimal *r*-value of 0.30 (Figure 1A), multiplied by the SD of the fMRI time series, τ = 1 and fMRI data lengths of 128, 120, 110, 100, 90, 95, and 85. Only data lengths where all 20 subjects returned SampEn values were included in the study. Data lengths less than 85 could not be included in the study because some of the subjects did not return SampEn values as a result of a lack of templates to compare. Whole brain SampEn maps were generated on a voxel by voxel basis using the same approach as Sokunbi et al. (2011) on a MATLAB and C platform. A threshold of 0.1 times the maximum signal was used to exclude voxels being calculated outside the brain. The mean whole brain SampEn value for each subject was computed. Also, the ROC area for discriminating between both groups was computed from the mean whole brain SampEn value of each subject in both groups for all the data lengths. SampEn showed good discriminating ability for 85 ≤ *N* ≤ 128 as shown in Figure 1B.

Similarly, whole brain SampEn maps were generated for the cohort of 43 younger and 43 elderly adults using *m* = 2, the optimal *r*-value of 0.30 (Figure 1A), multiplied by the SD of the fMRI time series, τ = 1 and fMRI data lengths of 128 and 85. The ROC area for discriminating between the cohort of 43 younger and 43 elderly adults was computed from the mean whole brain SampEn value of each subject in both groups for data lengths *N* = 128 and *N* = 85.

### Statistical analysis

The ROC analyses were performed on the mean whole brain SampEn values using the International Business Machines Corporation (IBM) Statistical Package for Social Sciences (SPSS 20.0; New York, USA) software. Independent *t*-tests for the different data lengths, *N*, were performed between the mean whole brain SampEn values of both groups using SPSS software. Also, correlations using the Pearson correlation analyses between the mean whole brain SampEn and age for the whole population were performed in SPSS, for the different data lengths, *N*. False discovery rate (FDR) for multiple comparisons correction (*q* < 0.05) in R-Statistics (http://www.r-project.org/) was used to correct the *p*-values of the independent *t*-tests and *p*-values of the Pearson's correlation analyses. The Pearson's correlation coefficients (*r*-values) were interpreted using Dancey and Reidy's categorisation (Dancey and Reidy, 2004). Here, *r*-value of ±1 is interpreted as a perfect correlation, *r*-values between ±0.7 to ±0.9 are interpreted as strong correlations, *r*-values in the range ±0.4 to ±0.6 are categorized as moderate correlations, *r*-values between ±0.1 to ±0.3 are weak correlations and an *r*-value of 0 is zero correlation, implying there is no correlation.

The SampEn map of each subject was normalized to a standard echo planar imaging (EPI) template, and a regional (spatial) analysis was performed using the two-sample *t*-test in SPM8, comparing the SampEn maps of the younger and elderly adults at a family-wise error (FWE) corrected cluster level significance of *p* < 0.05 and threshold *p* = 0.005. This was only done for data lengths *N* = 85 and *N* = 128. Correlations between the SampEn maps and age for the whole population were tested using multiple regression approach in SPM8.

## Results

### Small group of 10 younger and 10 elderly adults

**Figure 2:**
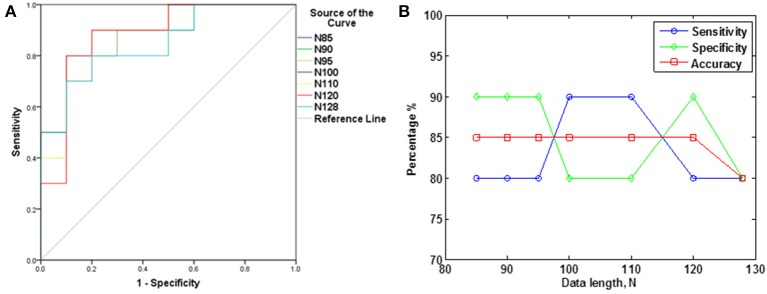
**ROC analyses portraying SampEn discriminatory characteristics for all the data lengths in the small group (A) ROC curves for 85 ≤ *N* ≤ 128. (B)** Plot of Sensitivity, Specificity and Accuracy against *N*.

**Figure 3:**
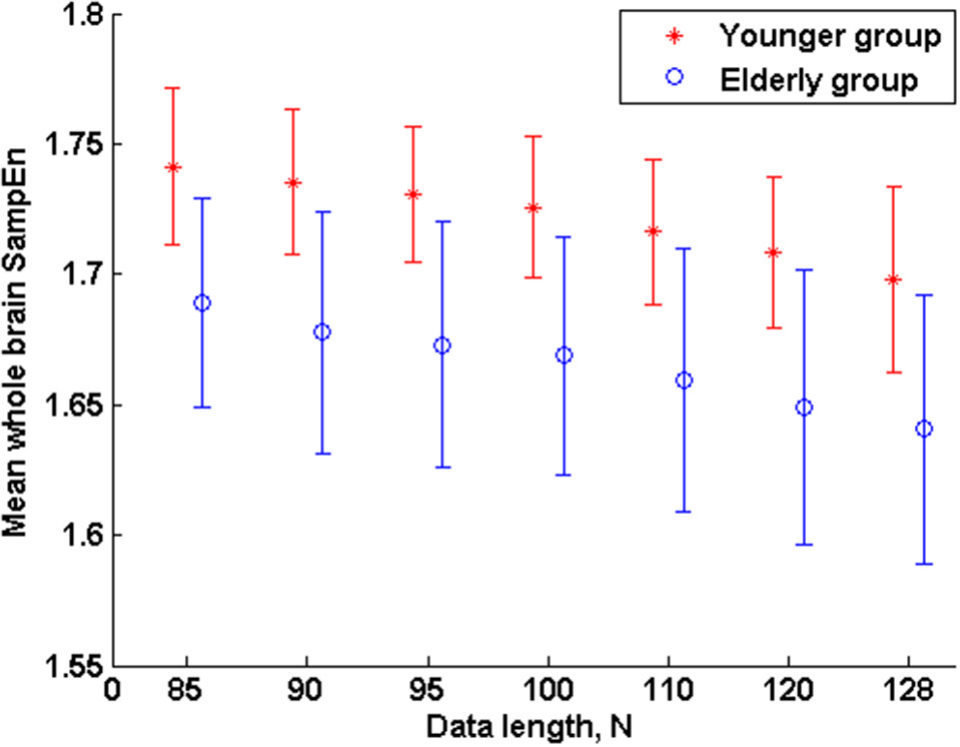
**Mean whole brain SampEn (*m* = 2, *r* = 0.30, 85 ≤ *N* ≤ 128) difference between younger and elderly adults for data length *N* in the small group analysis**. Here, the mean whole brain SampEn values of the younger adults were significantly (*p* < 0.05) higher than the mean whole brain SampEn values of the elderly adults.

**Figure 4:**
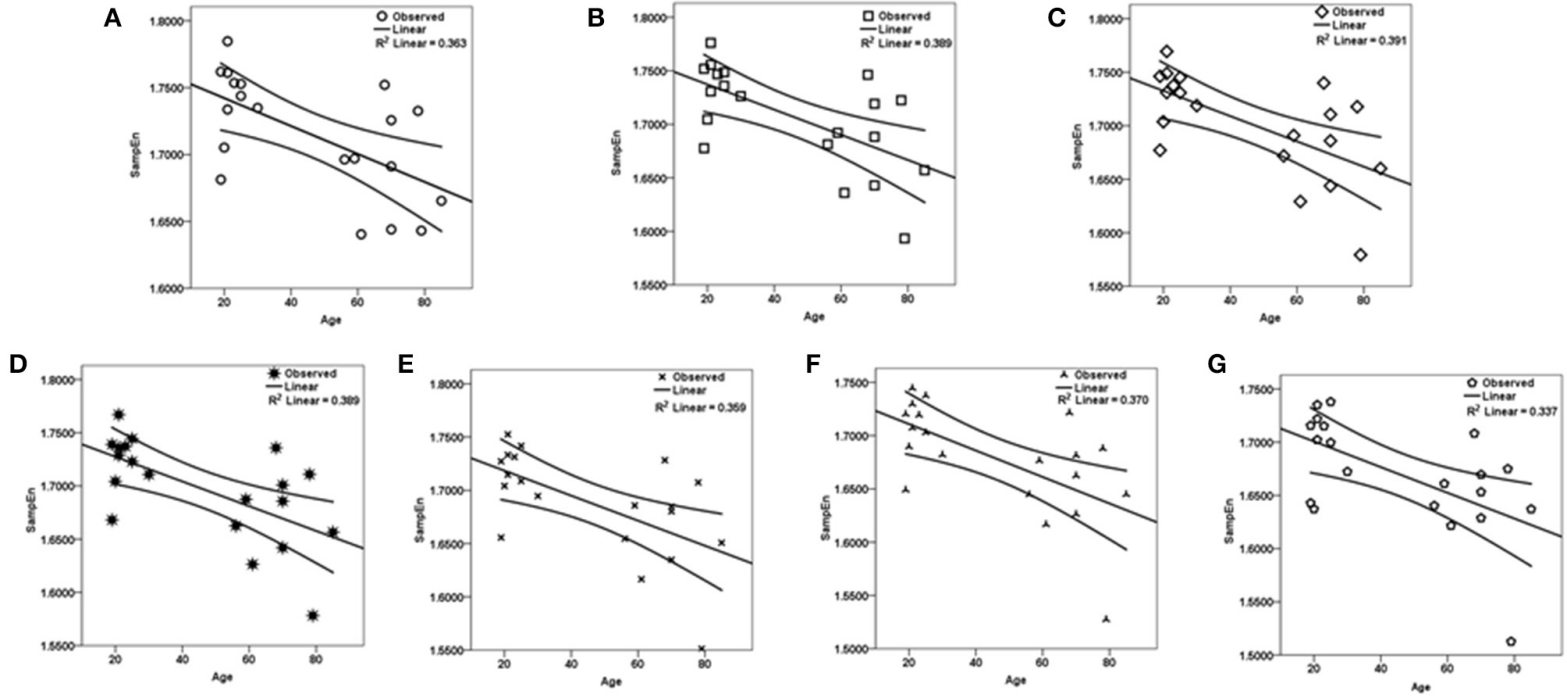
**Regression curve estimation between SampEn (*m* = 2, *r* = 0.30, 85 ≤ *N* ≤ 128) and age for all the data lengths *N* in the small group analysis**. SampEn of the population decrease with an increase in age, for all data lengths. **(A)** *N* = 85, **(B)** *N* = 90, **(C)** *N* = 95, **(D)** *N* = 100, **(E)** *N* = 110, **(F)** *N* = 120, **(G)** *N* = 128.

**Figure 5:**
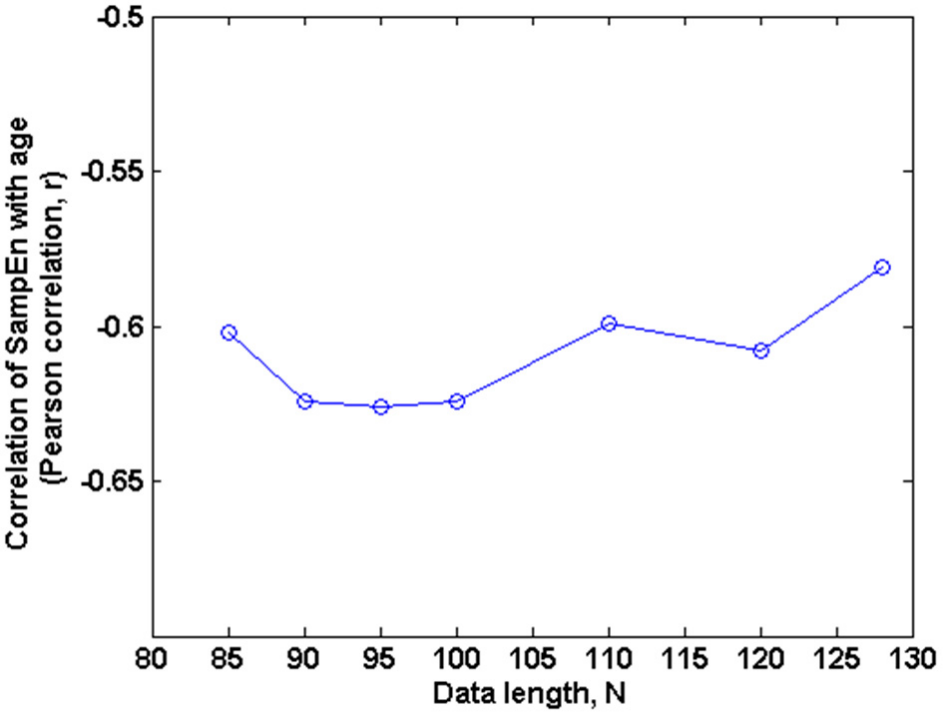
**Correlation of SampEn (*m* = 2, *r* = 0.30, 85 ≤ *N* ≤ 128) with age against *N* in the small group analysis**. Here, the Pearson's correlation coefficient is relatively constant with changing data length *N*.

The subjects' characteristics and SampEn measures for the small group of 10 younger and 10 elderly adults are shown in Table 1. The ROC results of the mean whole brain SampEn for data lengths 85 ≤ *N* ≤ 128 were in the range 0.850–0.890. This implies that the ability of SampEn to effectively discriminate the younger from the elderly adults across all the data lengths is good and that this ability is not dependent on data length. The ROC curves and characteristics for 85 ≤ *N* ≤ 128 are shown in Figure 2A. The sensitivity and specificity obtained from the ROC analysis ranged between 80 and 90% for all the data lengths, while the accuracy was 85% for all data lengths except for *N* = 128 where the accuracy dropped to 80% (see Figure 2B and Table 2). For data lengths 85 ≤ *N* ≤ 128, the mean whole brain SampEn values of the younger adults were significantly (*p* < 0.05) higher than the mean whole brain SampEn values of the elderly adults. After corrections for multiple comparisons using the FDR, the mean whole brain differences for all the data lengths remained significantly (*q* < 0.05) higher. The mean whole brain differences between the younger and elderly adults for all the data lengths are shown in Figure 3. Moderate negative correlations (*r*-values between −0.581 and −0.626) were obtained at *p* < 0.01 between the mean whole brain SampEn values and the age of the population, for all the data lengths (85 ≤ *N* ≤ 128). Also, after corrections for multiple comparisons using FDR, the moderate negative correlations between the mean whole brain SampEn values and age remained significant (*q* < 0.05). This implies that for all the data lengths SampEn decreased with age. Table 3 shows the Pearson's correlation coefficients, *r*, the *p*-values and the *q*-values (FDR) for data lengths 85 ≤ *N* ≤ 128. Figures 4A–G shows the regression curve estimation between SampEn and age for the population. A graph was plotted to further investigate how the Pearson's correlation coefficients, *r* (correlation of SampEn and age) varied with the different data lengths 85 ≤ *N* ≤ 128. The graph shown in Figure 5 shows that the Pearson's correlation coefficients, *r* remained relatively constant with the different data lengths. This implies that the correlation between SampEn and age was relatively consistent with the changes in data length.

**Table 1:** **Subjects' characteristics and SampEn measures for the small group of 10 younger and 10 elderly adults**.

	**Younger adults**	**Elderly adults**	**Significance (*p*-values)**	**Significance FDR corrected (*q*-values)**
Age (years)	22.40 ± 3.44	69.60 ± 9.25	*p* < 0.001	
Sex (M/F)	5/5	5/5		
SampEn at *N* = 85	1.7413 ± 0.0298	1.6888 ± 0.0400	*p* = 0.004	*q* = 0.007
SampEn at *N* = 90	1.7354 ± 0.0280	1.6779 ± 0.04631	*p* = 0.003	*q* = 0.007
SampEn at *N* = 95	1.7309 ± 0.0260	1.6729 ± 0.0472	*p* = 0.003	*q* = 0.007
SampEn at *N* = 100	1.7258 ± 0.0268	1.6687 ± 0.0458	*p* = 0.003	*q* = 0.007
SampEn at *N* = 110	1.7164 ± 0.0278	1.6595 ± 0.0506	*p* = 0.006	*q* = 0.007
SampEn at *N* = 120	1.7082 ± 0.0288	1.6489 ± 0.0529	*p* = 0.006	*q* = 0.007
SampEn at *N* = 128	1.6980 ± 0.0359	1.6407 ± 0.0517	*p* = 0.010	*q* = 0.010

**Table 2:** **ROC characteristics for the small group of 10 younger and 10 elderly adults**.

**Data length, *N***	**Threshold**	**Sensitivity (%)**	**Specificity (%)**	**Accuracy (%)**	**Area under the ROC curve**
85	1.7332	80	90	85	0.880
90	1.7244	80	90	85	0.880
95	1.7183	80	90	85	0.880
100	1.7026	90	80	85	0.890
110	1.6902	90	80	85	0.880
120	1.6888	80	90	85	0.880
128	1.6710	80	80	80	0.850

**Table 3:** **Correlation of SampEn with age for the small group of 10 younger and 10 elderly adults**.

	**Pearson's correlation (*r*-values)**	**Significance (*p*-values)**	**Significance FDR corrected (*q*-values)**
SampEn at *N* = 85	−0.602	*p* = 0.005	*q* = 0.006
SampEn at *N* = 90	−0.624	*p* = 0.003	*q* = 0.006
SampEn at *N* = 95	−0.626	*p* = 0.003	*q* = 0.006
SampEn at *N* = 100	−0.624	*p* = 0.003	*q* = 0.006
SampEn at *N* = 110	−0.599	*p* = 0.005	*q* = 0.006
SampEn at *N* = 120	−0.608	*p* = 0.004	*q* = 0.006
SampEn at *N* = 128	−0.581	*p* = 0.007	*q* = 0.007

**Figure 6:**
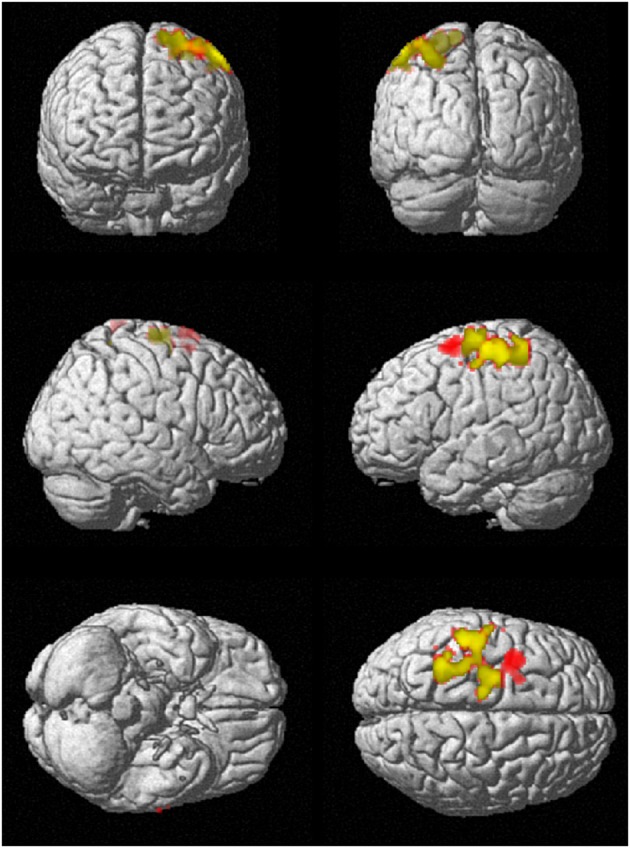
**SampEn (*m* = 2, *r* = 0.30, *N*) differences between younger and elderly adults for the small group analysis**. *N* = 85 is red and *N* = 128 is green. Overlap is yellow. SampEn values of the younger adults were significantly (*p* < 0.05) higher than SampEn values of the elderly adults with the corresponding brain regions as shown.

**Figure 7:**
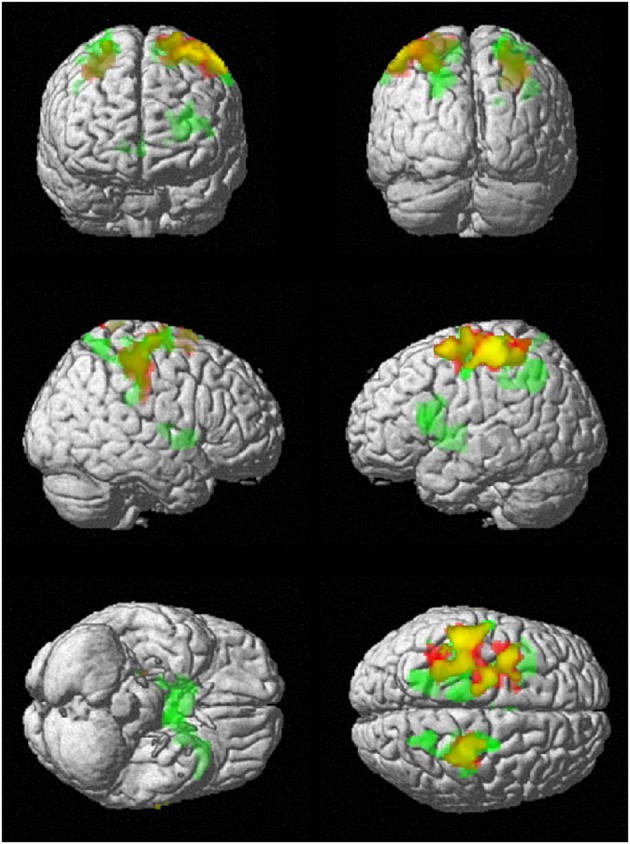
**Correlation of SampEn (*m* = 2, *r* = 0.30, *N*) with age for the small group analysis**. *N* = 85 is red and *N* = 128 is green. Overlap is yellow. SampEn for the population decrease as age increase with corresponding brain regions as depicted.

To investigate regional differences and similarities in data lengths, the whole brain SampEn maps for the minimum and maximum data lengths (85 ≤ *N* ≤ 128) were tested regionally with a family-wise error (FWE) corrected cluster level significance of *p* < 0.05 using the two-sample *t*-test in SPM8. The results consistent with that of the mean whole brain analysis show that the younger adults exhibited significantly (*p* < 0.05) higher SampEn values than the elderly adults at a threshold of *p* = 0.005 with corresponding discriminated brain regions. For data length *N* = 85, only the frontal lobe of the brain was discriminated while for *N* = 128, the frontal lobe and parietal lobe were discriminated. These discriminated brain regions are listed in Table 4. Figure 6 shows the rendered images of the two-sample *t*-tests between the younger and elderly adults, for data lengths, *N* = 85 and *N* = 128. Also, correlations between the whole brain SampEn maps and age, of the whole population, for data lengths, *N* = 85 and *N* = 128 were performed using multiple regression analysis in SPM8. Again, SampEn portrayed a significant (*p* < 0.05) negative correlation with age, for both data lengths as shown by the rendered images in Figure 7. For *N* = 85, the frontal, limbic and parietal lobes were discriminated while for *N* = 128 the frontal lobe, limbic lobe, parietal lobe and sub-lobar brain regions were discriminated. See Table 5 for a list of the discriminated brain regions.

**Table 4:** **SampEn differences for the small group of 10 younger and 10 elderly adults**.

**Data length, *N***	**Cluster number and extent**	**Brain region**	**Talairach coordinate (*X, Y, Z*)**	**Brain label**	**Tissue type**	**Cluster *p*-value (FWE corrected)**	**Voxel *t*-value**
85	Cluster 1 Extent = 2181	Frontal lobe	−34, 2, 66	Left middle frontal gyrus	Gray matter	*p* < 0.001	5.47
128	Cluster 1 Extent = 889	Frontal lobe	−22, −14, 66	Left middle frontal gyrus	Gray Matter	*p* = 0.007	4.02
		Parietal lobe	−28, −44, 56	Left inferior parietal lobule	White matter	*p* = 0.007	4.26
		Parietal lobe	−46, −22, 60	Left post-central gyrus	Gray matter	*p* = 0.007	3.90

Location coordinates are those of the peak significance in each region (threshold p = 0.005, FWE corrected cluster p < 0.05).

**Table 5:** **SampEn correlation with age for *N* = 85 and *N* = 128, for the small group of 10 younger and 10 elderly adults**.

**Data length, *N***	**Cluster number and extent**	**Brain region**	**Talairach coordinate (*X, Y, Z*)**	**Brain label**	**Tissue type**	**Cluster *p*-value (FWE corrected)**	**Voxel *t*-value**
85	Cluster 1 Extent = 768	Frontal lobe	36, −22, 48	Right post-central gyrus	White matter	*p* = 0.015	6.96
		Frontal lobe	30, −22, 38	Right sub-gyral	White matter	*p* = 0.015	5.30
		Limbic lobe	20, −24, 40	Right cingulate gyrus	White matter	*p* = 0.015	5.14
	Cluster 2 Extent = 3320	Frontal lobe	−34, 2, 66	Left middle frontal gyrus	Gray matter	*p* < 0.001	5.69
		Parietal lobe	−46, −22, 60	Left post-central gyrus	Gray matter	*p* < 0.001	5.42
128	Cluster 1 Extent = 1247	Frontal lobe	−30, 16, 16	Left sub-gyral	White matter	*p* = 0.004	8.40
		Limbic lobe	2, 2, −4	Right anterior cingulate	Gray matter	*p* = 0.004	5.28
		Sub-lobar	−6, −2, 4	Left extra-nuclear	White matter	*p* = 0.004	5.26
	Cluster 2 Extent = 3406	Parietal lobe	−26, −42, 56	Left sub-gyral	White matter	*p* < 0.001	5.90
		Parietal lobe	−20, −54, 40	Left pre-cuneus	White matter	*p* < 0.001	5.32
		Parietal lobe	−50, −28, 58	Left post-central gyrus	Gray matter	*p* < 0.001	4.85
	Cluster 3 Extent = 1246	Parietal lobe	32, −34, 54	Right post-central gyrus	Gray matter	*p* = 0.004	5.09
		Frontal lobe	20, −18, 64	Right middle frontal gyrus	White matter	*p* = 0.004	4.65
		Parietal lobe	28, −28, 48	Right sub-gyral	White matter	*p* = 0.004	4.48

Location coordinates are those of the peak significance in each region (threshold p = 0.005, FWE corrected cluster p < 0.05).

### Cohort of 43 younger and 43 elderly adults

The subjects' characteristics and SampEn measures for the whole ICBM resting state cohort of 43 younger and 43 elderly adults are shown in Table 6. The ROC results of the mean whole brain SampEn for data lengths *N* = 85 and *N* = 128 were 0.600 and 0.603 respectively. This implies that the ability of SampEn to effectively discriminate the younger from the elderly adults of both data lengths is poor. For data length *N* = 85, the sensitivity was 65.10%, the specificity was 53.50% and accuracy was 59.30% at a threshold of 1.7298. While for data length *N* = 128, the sensitivity was 58.10%, the specificity was 58.10% and accuracy was 58.10% at a threshold of 1.6986. For both data lengths, the mean whole brain SampEn values of the younger and elderly adults were not significantly (*p* > 0.05) different but the younger adults had higher mean whole brain SampEn values than the elderly adults. Weak negative correlations, *r*-values of −0.078 and −0.099 were obtained at *p* > 0.05 between the mean whole brain SampEn values and the age of the population, for data lengths *N* = 85 and *N* = 128 respectively.

**Table 6:** **Subjects' characteristics and SampEn measures for the whole ICBM resting state cohort of 43 younger and 43 elderly adults**.

	**Younger adults**	**Elderly adults**	**Significance (*p*-values)**
Age (years)	29.05 ± 8.66	59.33 ± 10.27	*p* < 0.001
Sex (M/F)	21/22	20/23	
SampEn at *N* = 85	1.7387 ± 0.0526	1.7172 ± 0.0597	*p* = 0.080
SampEn at *N* = 128	1.6979 ± 0.0545	1.6735 ± 0.0655	*p* = 0.065

For data length, *N* = 128, the result of the regional analysis show that the younger adults exhibited higher SampEn values than the elderly adults at a threshold of *p* = 0.005 with a family-wise error (FWE) corrected cluster level significance of *p* < 0.05 at the parietal and frontal lobes. These discriminated brain regions are listed in Table 7. For data length, *N* = 85, the younger adults also exhibited higher SampEn values than the elderly adults at the left parietal lobe (−24, −48, 54, Sub-Gyral, White Matter; −22, −52, 44, Precuneus, White Matter; −32, −40, 52, Postcentral Gyrus, White Matter) with a threshold of *p* = 0.005 and at an uncorrected *p*-value of 0.005. When the analysis at *N* = 85 was corrected for multiple comparisons, the discriminated brain region was not significant (*p* > 0.05). There were no significant (*p* > 0.05) correlations between the whole brain SampEn maps and age, of the whole population, for both data lengths (*N* = 85 and *N* = 128).

**Table 7:** **SampEn differences for the whole ICBM resting state cohort of 43 younger and 43 elderly adult**.

**Data length, *N***	**Cluster number and extent**	**Brain region**	**Talairach coordinate (*X, Y, Z*)**	**Brain label**	**Tissue type**	**Cluster *p*-value (FWE corrected)**	**Voxel *t*-value**
128	Cluster 1 Extent = 2251	Parietal lobe	−24, −46, 56	Left sub-gyral	Gray matter	*p* < 0.001	4.41
		Parietal lobe	−24, −56, 52	Left precuneus	White matter	*p* < 0.001	3.58
		Parietal lobe	−46, −22, 60	Left inferior parietal lobule	Gray matter	*p* < 0.001	3.11
		Frontal lobe	−26, −30, 66	Left precentral gyrus	Gray matter	*p* < 0.001	3.00
		Frontal lobe	−28, −24, 46	Left sub-gyral	White matter	*p* < 0.001	2.95

Location coordinates of the significant regions (threshold p = 0.005, FWE corrected cluster p < 0.05).

**Figure 8:**
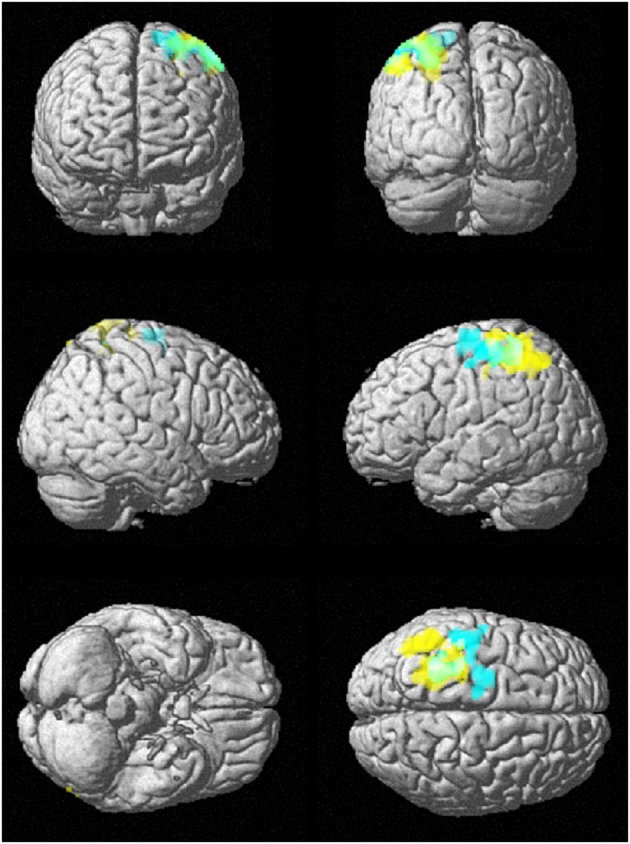
**SampEn (*m* = 2, *r* = 0.30, 128) differences between younger and elderly adults for the small group (10 younger and 10 elderly adults) and the whole cohort (43 younger and 43 elderly adults)**. Small group is cyan, whole cohort is yellow, and overlap is green. SampEn values of the younger adults were significantly (*p* < 0.05) higher than SampEn values of the elderly adults with the corresponding brain regions as shown.

Figure 8 shows the rendered images of the two-sample *t*-tests between the younger and elderly adults, for the small group (10 younger and 10 elderly adults) and the whole cohort (43 younger and 43 elderly adults) at data length *N* = 128. The images show that both analyses had overlapping discriminated brain regions between the frontal and parietal lobes.

## Discussion

The aim of this study was to test the ability of SampEn to effectively discriminate between two different age groups of resting state fMRI data with data length, *N* less than 10^*m*^ (where *m* = 2). For the small group analysis, the results of the whole brain analyses shows that the ROC areas for *N* = 85, 90, and 95 were the same (0.880), the ROC area for *N* = 100 was 0.890, the areas for *N* = 110 and 120 were 0.880, and for *N* = 128 was 0.850. The disproportionality of these ROC areas to the respective data lengths is in line with the notion that SampEn is largely independent of data length. Furthermore, the same level of accuracy (85%) exhibited by all the data lengths with the exception of *N* = 128 having accuracy of 80%, indicates that SampEn displays some relative consistency. Also, the mean whole brain SampEn of the younger adults was significantly (*p* < 0.05) higher than the elderly adults across data lengths, 85 ≤ *N* ≤ 128. There were also moderate negative correlations (*r*-values between −0.581 and −0.626) (see Table 3) between the mean whole brain SampEn values and age for 85 ≤ *N* ≤ 128 at *q* < 0.05. Wang et al. (2014) showed that data length has only a minor effect on SampEn, which ensured including all the resting state fMRI data at the 1000 Functional Connectomes project repository, even with different time points for their brain entropy (BEN) mapping.

In the regional analyses of the small group, the younger adults exhibited significantly higher SampEn than the elderly adults, only at the frontal lobe for *N* = 85, and at the frontal and parietal lobes for *N* = 128. For *N* = 85, there was a significant negative correlation between SampEn and age at the frontal, limbic and parietal lobes while for *N* = 128, this negative correlation occurred at the frontal lobe, limbic lobe, parietal lobe and sub-lobar region. These associations indicate that there is reduction in entropy with increase in age. This reduction in entropy is common to both analyses (at *N* = 85 and *N* = 128), independent of the different data lengths and overlaps at the frontal, limbic and parietal lobes of the brain. The frontal lobe has been implicated in age-related processes resulting in a decline in memory functions (Craik and Salthouse, 2000). In a diffusion tensor imaging (DTI) study of a healthy population of 25–70 years, the limbic system which is responsible for emotion processing and memory function has been shown to undergo degradation with ageing (Gunbey et al., 2014). The sub-lobar brain region has been implicated in white matter structures associated with cognitive ageing (Staff et al., 2006). Also, decreased fractional anisotropy (FA) measurements in the frontal and parietal lobes has been associated with poorer cognitive performance in a study investigating the relationship between FA and selected measures of cognition across a broad age group (20–73 years of healthy subjects) to explore a possible structural basis for cognitive changes with age (Grieve et al., 2007). Our findings of decrease in entropy with age are consistent with Lipsitz's (2004) entropy definition of ageing (loss of entropy) and Vaillancourt and Newell's (2002) fixed-point attractor postulate where complexity decreases with age and disease.

Comparing the whole cohort (43 younger and 43 elderly adults) to the small group (10 younger and 10 elderly adults) analysis at data lengths *N* = 85 and 128, the small group analysis discriminated between the younger and elderly adults, and showed that the fMRI brain complexity decreases with age at both data lengths. The whole cohort analysis only discriminated between the younger and elderly adults at *N* = 128. The whole cohort analysis at *N* = 85 was indicative of the ageing process but this indication was not significant (*p* > 0.05). The inability of SampEn to portray the same discriminatory effect for both the small group and whole cohort analyses may be due to two factors. Firstly, it may be due to the variance in the heterogeneous distribution of the subjects' ages in both datasets. For the small group, the mean age of the younger and elderly adults is (22.40 ± 3.44) and (69.60 ± 9.25) respectively, while in the whole cohort the mean age of the younger and elderly adults is (29.05 ± 8.66) and (59.33 ± 10.27) respectively. Clearly, there is disparity in the mean and SD of the younger and elderly adults between the small group and whole cohort. The second factor may be due to the limited discriminatory ability of SampEn. This study was conducted with SampEn on a single scale, a multiscale SampEn analysis is superior to a single scale analysis and portrays a superior discriminatory ability (Costa et al., 2002; Yang et al., 2013). Another approach which may show superior discriminatory ability to SampEn is single scale Fuzzy approximate entropy (fApEn) (Xie et al., 2010), which has not been investigated in comparison to SampEn and in fMRI datasets.

An increase in functional entropy with age (Yao et al., 2013) was found in a recent study, where Shannon entropy; a measure of information, choice and uncertainty (in bits) (Shannon, 1948) was used as a bivariate measure to characterize the correlation coefficient (considered as a random variable) of a distinct pair of brain regions. The resulting entropy measure in bits was called functional entropy. The functional entropy measured the dispersion (or spread) of functional connectivity that exists within the brain. At the population level, they found that the functional entropy of the human brain increases with age where a higher level of randomness reflected the way different brain-regions functionally interacted with one another. At the regional level, they found some regions where the functional entropy increases, decreases and where it remains almost constant. They noted a decrease in functional entropy with age in the left and right insulars. Furthermore, a computational model based on DTI was used to investigate the origins of the relationship between functional entropy and age. The model implicated a brain entropy that decreases when the excitatory connection strength and neuron number in each brain region are simultaneously reduced. In the present study, our analysis entailed a univariate characterization of a voxel with SampEn. Here, SampEn is used as an estimate of complexity and returns a dimensionless numerical value. Our results showed that sample entropy decrease with age. SampEn and ApEn are not the same as Shannon entropy, they are used to indicate system complexity because both of them were defined as approximates to the Kolmogorov complexity (Wang et al., 2014).

In the small group analysis, the reduction in the accuracy of SampEn to effectively discriminate the younger from the elderly adults (in the mean whole brain analyses) from 85% for data lengths 85 ≤ *N* ≤ 120 to 80% for data length *N* = 128 may be attributed to the “averaging effect” which is basically the simplest form of a digital filter and is a means of reducing the effect of random noise (Smith, 1999). Averaging the BOLD fMRI response of a voxel over a number of data lengths can help to improve the BOLD signal to noise ratio. The amount of noise reduction that this “averaging effect” can produce is equal to the square-root of the data length in the average (Smith, 1999). For example, data lengths of *N* = 85, 90, 95, 100, 110, 120, and 128 of BOLD fMRI signal would reduce the noise by a factor of 9.22, 9.49, 9.75, 10.00, 10.49, 10.96, and 11.31 respectively. As a result of this, the level of noise in data length *N* = 128 is less compared to data length *N* = 85 and vice versa. The level of noise in data length *N* = 85 is higher than *N* = 128. Since noise is the signal with the most complex dynamics and highest measured entropy (Lu et al., 2008), it is expected that the entropy of the younger and elderly adults for data length *N* = 85 would be higher than the corresponding groups in data length *N* = 128 and was therefore reflected in the measured accuracies. This is evident in the mean whole brain SampEn measurements for 85 ≤ *N* ≤ 128 in Table 1. Here it can be clearly seen that the measured SampEn values decreases as the data length increases from *N* = 85 to *N* = 128, implying that the level of noise decrease from *N* = 85 to *N* = 128. Another obvious evidence suggesting the influence of noise in the accuracy was demonstrated in the regional analyses where noise played an opposite effect. Here, Sample entropy discriminated more brain regions at *N* = 128 than *N* = 85. The difference in the discriminated brain regions can be attributed to the influence of a higher noise level in *N* = 85 than *N* = 128. Sample entropy (an optimized approximate entropy) is nearly unaffected by low level noise, is robust to occasional very large or small artifacts, gives meaningful information with a reasonable number of data lengths, and is finite for both stochastic and deterministic processes (Zhang and Roy, 2001).

In the computation of Sample entropy from an fMRI signal, a high noise level is a potential confounder and may prevent Sample entropy from discriminating effectively between system complexities. The noise present in fMRI data consists of system noise (white noise), arising from both thermal noise and hardware imperfections, and 1/f low-frequency noise, physiological fluctuations from respiratory and cardiac activities. The noise level can be reduced as we have done by applying high pass filtering to reduce the low frequency components of the noise and spatial smoothing to reduce the system noise. With the level of noise reduced, an optimized and robust computation of Sample entropy can be implemented with an appropriate tolerance value, *r*. To avoid a significant contribution from noise in the calculation of the entropy, one must choose *r* larger than most of the noise (Pincus, 1991). A higher *r*-value shows better robustness to reduced noise in distinguishing the nonlinear system dynamics (Xie et al., 2010) of the experimental and control groups. When a small *r*-value is used, the algorithm identifies two sections being compared as dissimilar when the difference may be brought about by noise. Using a larger *r* avoids the misclassification. Using a large *r*, however, may result in some signal detail being lost. The selection of the appropriate *r* is essentially a compromise between these two phenomena: i.e., an *r* large enough that allows the Sample entropy algorithm to distinguish the system signal from noise, but small enough to allow the algorithm to assess the detail present in the signal (Chen et al., 2009). We have used a higher *r*-value to obtain an optimized and robust computation of Sample entropy in the presence of minimal noise. The *r*-value (*r* = 0.30) we used showed better robustness to reduced noise in distinguishing the nonlinear system dynamics of both younger and elderly adults (Figure 1A).

Some studies have suggested that the bias of SampEn from short data lengths may be compensated for by using a small pattern length (*m* = 1) and a relatively large similarity factor (tolerance value), *r*, to accommodate the short and noisy BOLD data (Yang et al., 2013). The choice of *m* = 2 is superior to *m* = 1 because it allows more detailed reconstruction of the joint probabilistic dynamics of the time series (Pincus and Goldberger, 1994). It has also been shown that using *m* = 2 is more consistent than *m* = 1 over a wider range of tolerance values, *r* (Sokunbi et al., 2013). Using *m* = 2 implies that the SampEn of fMRI data with data length less than 100 can be computed with robust and optimized parameter contrary to the suggestion of others (Abasolo et al., 2006; Yang et al., 2013), avoiding erroneous data length constraint. Also, *m* = 2 has been used for data length *N* = 50 of i.i.d uniform random numbers (Chen et al., 2009).

Richman and Moorman (2000) concluded that the SampEn (*m*, *r*, *N*) statistics are not completely unbiased under all conditions. They found that the bias of SampEn was less than 3% for data lengths greater than 100 but as high as 35% for data length of 15 points and that the bias of SampEn for very small data sets is largely due to non-independence of templates. They suggested that one method of removing this bias would be to partition the time series but noted that this unbiased approach has the potentially severe limitation of reducing the number of possible template matches and enlarging the confidence intervals about the SampEn estimate. They also argue that because this bias appears to be present only for very small *N*, the disjoint template approach does not appear necessary in usual practice. One notable limitation of the present study is that we would expect the bias of our fMRI SampEn (2, 0.30, 85 ≤ *N* ≤ 128) analyses to be in the proximity of the bias of less than 3% for data lengths greater than 100. Another limitation of SampEn is that SampEn values for data lengths less than 85 could not be obtained because of a lack of templates to compare.

## Conclusion

The small group fMRI SampEn analyses provided additional evidence that it is possible to obtain good discriminating feature from fMRI data with data lengths less than 100, indicating that SampEn is largely independent on changes in data length and displays some relative consistency. While it is better to acquire data with longer data lengths for best analysis results, low noise level and minimum bias, it is not always possible to do this with fMRI data because of the nature of some fMRI experiments and its temporal limitation. SampEn is a possible analysis tool amongst time series analysis techniques because it is less sensitive to changes in data length and relatively consistent. SampEn is well suited for short data sets like fMRI data, though a compromise has to be made with the increase in noise level as data length decreases. The heterogeneous distribution of the subjects ages in the whole cohort ages compared to the small group ages may have limited the single scale discriminatory ability of SampEn in the whole cohort analyses. A multiscale SampEn analysis may portray a superior discriminatory ability. In the present study, using *m* = 2 ensures that SampEn is computed for fMRI data (having data length less than 100) with robust and optimized parameter thereby avoiding the erroneous data length constraint of 10^*m*^–20^*m*^. Finally, before characterizing data sets, especially short data sets with SampEn, we would recommend using optimal parameters; an *m* of 2 or as appropriate and to determine the *r*-value (by examining several *r*-values) where SampEn displays its best discriminating ability.

### Conflict of interest statement

The author declares that the research was conducted in the absence of any commercial or financial relationships that could be construed as a potential conflict of interest.

## References

[B1] Abasolo D. Hornero R. Espino P. Álvarez D. Poza J. (2006). Entropy analysis ofthe EEG background activity in Alzheimer's disease patients. Physiol. Meas. 27, 241–253 10.1088/0967-3334/27/3/003 16462011

[B2] Chen W. Zhuang J. Yu W. Wang Z. (2009). Measuring complexity using FuzzyEn, ApEn, and SampEn. Med. Eng. Phys. 31, 61–68 10.1016/j.medengphy.2008.04.005 18538625

[B3] Costa M. Goldberger A. L. Peng C. K. (2002). Multiscale entropy analysis of complex physiologic time series. Phys. Rev. Lett. 89:068102 10.1103/PhysRevLett.89.068102 12190613

[B4] Craik F. I. M. Salthouse T. A. (eds.). (2000). The Handbook of Aging and Cognition. 2nd Edn New Jersey: Lawrence Erlbaum Associates, Inc

[B5] Dancey C. Reidy J. (2004). Statistics Without Maths for Psychology: Using SPSS for Windows. London: Prentice Hall

[B6] Deary I. J. Corley J. Gow A. J. Harris S. E. Houlihan L. M. Marioni R. E. (2009). Age-associated cognitive decline. Br. Med. Bull. 92, 135–152 10.1093/bmb/ldp033 19776035

[B7] Eckmann J. P. Ruelle D. (1992). Fundamental limitations for estimating dimensions and Lyapunov exponents in dynamical system. Physica D 56, 185–187 10.1016/0167-2789(92)90023-G

[B8] Ferenets R. Vanluchene A. Lipping T. Heyse B. Struys M. M. (2007). Behavior of entropy/complexity measures of the electroencephalogram during propofol-induced sedation: dose-dependent effects of remifentanil. Anesthesiology 106, 696–706 10.1097/01.anes.0000264790.07231.2d 17413907

[B9] Gaal Z. A. Boha R. Stam C. J. Molnár M. (2010). Age-dependent features of EEG- reactivity-Spectral, complexity, and network characteristics. Neurosci. Lett. 479, 79–84 10.1016/j.neulet.2010.05.037 20560166

[B10] Goldberger A. L. Peng C. Lipsitz L. A. (2002). What is physiologic complexity and how does it change with aging and disease? Neurobiol. Aging 23, 23–26 10.1016/S0197-4580(01)00266-4 11755014

[B11] Gomez C. Poza J. Garcia M. Fernandez A. Hornero R. (2011). Regularity analysis of spontaneous MEG activity in Attention-Deficit/Hyperactivity Disorder, in 33rd Annual International Conference of the IEEE EMBS (Boston, MA), 1765–1768 10.1109/IEMBS.2011.6090504 22254669

[B12] Grassberger P. Procaccia I. (1983). Characterization of strange attractors. Phys. Rev. Lett. 50, 346–349 10.1103/PhysRevLett.50.346

[B13] Grieve S. M. Williams L. M. Paul R. H. Clark C. R. Gordon E. (2007). Cognitive aging, executive function, and fractional anisotropy: a diffusion tensor MR imaging study. Am. J. Neuroradiol. 28, 226–235 17296985 PMC7977408

[B14] Gunbey H. P. Ercan K. Findikoglu A. S. Bulut H. T. Karaoglanoglu M. Arslan H. (2014). The limbic degradation of aging brain: a quantitative analysis with diffusion tensor imaging. Sci. World J. 2014:196513 10.1155/2014/196513 24977184 PMC4009154

[B15] Lipsitz L. A. (2004). Physiological complexity, aging, and the path to frailty. Sci. Aging knowledge Environ. 2004:pe16 10.1126/sageke.2004.16.pe16 15103055

[B16] Lu S. Chen X. Kanters J. K. Solomon I. C. Chon K. H. (2008). Automatic selection of the threshold value *r* for approximate entropy. IEEE Trans. Biomed. Eng. 55, 1966–1972 10.1109/TBME.2008.919870 18632359

[B17] Pincus S. (1995). Approximate entropy (ApEn) as a complexity measure. Chaos 5, 110–117 10.1063/1.166092 12780163

[B18] Pincus S. M. (1991). Approximate entropy as a measure of system complexity. Proc. Natl. Acad. Sci. U.S.A. 88, 2297–2301 10.1073/pnas.88.6.2297 11607165 PMC51218

[B19] Pincus S. M. (2001). Assessing serial irregularity and its implications for health. Ann. N.Y. Acad. Sci. 954, 245–267 10.1111/j.1749-6632.2001.tb02755.x 11797860

[B20] Pincus S. M. Goldberger A. L. (1994). Physiological time-series analysis: what does regularity quantify? Am. J. Physiol. 266, H1643–H1656 8184944 10.1152/ajpheart.1994.266.4.H1643

[B21] Pool R. (1989). Is it healthy to be chaotic? Science 243, 604–607 10.1126/science.2916117 2916117

[B22] Pritchard W. S. Duke D. W. Coburn K. L. Moore N. C. Tucker K. A. Jann M. W. (1994). EEG-based neural-net predictive classification of Alzheimer's disease versus control subjects is augmented by non-linear EEG measures. Electroencephalogr. Clin. Neurophysiol. 91, 118–130 10.1016/0013-4694(94)90033-7 7519141

[B23] Richman J. S. Moorman J. R. (2000). Physiological time-series analysis using approximate and sample entropy. Am. J. Physiol. 278, H2039–H2049 10843903 10.1152/ajpheart.2000.278.6.H2039

[B24] Shannon C. E. (1948). A mathematical theory of communication. Bell Syst. Tech. J. 27, 379–656 10.1002/j.1538-7305.1948.tb01338.x

[B25] Smith S. W. (1999). The Scientist and Engineer's Guide to Digital Signal Processing. 2nd Edn San Diego, CA: California Technical Publishing

[B26] Sokunbi M. O. Fung W. Sawlani V. Choppin S. Linden D. E. J. Thome J. (2013). Resting state fMRI entropy probes complexity of brain activity in adults with ADHD. Psychiatry Res. 214, 341–348 10.1016/j.pscychresns.2013.10.001 24183857

[B27] Sokunbi M. O. Gradin V. B. Waiter G. D. Cameron G. G. Ahearn T. S. Murray A. D. (2014). Nonlinear complexity analysis of brain fMRI signals in schizophrenia. PLoS ONE 9:e95146 10.1371/journal.pone.0095146 24824731 PMC4019508

[B28] Sokunbi M. O. Staff R. T. Waiter G. D. Ahearn T. S. Fox H. C. Deary I. J. (2011). Inter-individual differences in fMRI entropy measurements in old age. IEEE Trans. Biomed. Eng. 58, 3206–3214 10.1109/TBME.2011.2164793 21859598

[B29] Staff R. T. Murray A. D. Deary I. J. Whalley L. J. (2006). Generality and specificity in cognitive aging: a volumetric brain analysis. Neuroimage 30, 1433–1440 10.1016/j.neuroimage.2005.11.004 16410052

[B30] Vaillancourt D. E. Newell K. M. (2002). Changing complexity in human behavior and physiology through aging and disease. Neurobiol. Aging 23, 1–11 10.1016/S0197-4580(01)00247-0 11755010

[B31] Wang Z. Li Y. Childress A. R. Detre J. A. (2014). Brain entropy mapping using fMRI. PLoS ONE 9:e89948 10.1371/journal.pone.0089948 24657999 PMC3962327

[B32] Wolf A. Swift J. B. Swinney H. L. Vastano J. A. (1985). Determining Lyapunov exponents from a time series. Physica D 16, 285–317 10.1016/0167-2789(85)90011-9

[B33] Xie H. B. Guo J. Y. Zheng Y. P. (2010). Fuzzy approximate entropy analysis of chaotic and natural complex systems: detecting muscle fatigue using electromyography signals. Ann. Biomed. Eng. 38, 1483–1496 10.1007/s10439-010-9933-5 20099031

[B34] Yao Y. Lu W. L. Xu B. Li C. B. Lin C. P. Waxman D. (2013). The increase of the functional entropy of the human brain with age. Nat. Sci. Rep. 3, 2853 10.1038/srep02853 24103922 PMC3793229

[B35] Yang A. C. Huang C. C. Yeh H. L. Liu M. E. Hong C. J. Tu P. C. (2013). Complexity of spontaneous BOLD activity in default mode network is correlated with cognitive function in normal male elderly: a multiscale entropy analysis. Neurobiol. Aging. 34, 428–438 10.1016/j.neurobiolaging.2012.05.004 22683008

[B36] Yentes J. M. Hunt N. Schmid K. K. Kaipust J. P. McGrath D. Stergiou N. (2013). The appropriate use of approximate entropy and sample entropy with short data sets. Ann. Biomed. Eng. 41, 349–365 10.1007/s10439-012-0668-3 23064819 PMC6549512

[B37] Zhang X. Roy R. J. (2001). Derived fuzzy knowledge model for estimating the depth of Anesthesia. IEEE Trans. Biomed. Eng. 48, 312–323 10.1109/10.914794 11327499

[B38] Zweig M. H. Campbell G. (1993). Receiver-operating characteristic (ROC) plots: a fundamental evaluation tool in clinical medicine. Clin. Chem. 39, 561–577 8472349

