# Supplementary material for: Sample entropy reveals high discriminative power between young and elderly adults in short fMRI data sets
Source: Front Neuroinform. 2014 Jul 23;8:69. doi: 10.3389/fninf.2014.00069 (PMC4107942; doi:10.3389/fninf.2014.00069)
Supplement: Supplementary file 1 [file DataSheet1.DOCX]

**Supplementary data**

**Table S1: Subject’s demographics used in the small group analysis**

| **No** | **Subject Code** | **Age** | **Sex^a^** | **Handedness^b^** | **Group** |
| --- | --- | --- | --- | --- | --- |
| 1  2  3  4  5  6  7  8  9  10  11  12  13  14  15  16  17  18  19  20 | sub13384  sub28808  sub65921  sub55656  sub71932  sub98317  sub09539  sub00448  sub47753  sub82754  sub98802  sub19395  sub29353  sub73490  sub02503  sub22674  sub68850  sub87217  sub48830  sub93262 | 19  19  20  21  21  21  23  25  25  30  56  59  61  68  70  70  70  78  79  85 | f  m  f  f  m  f  f  m  m  m  m  f  m  f  m  m  m  f  f  f | R  R  R  R  R  R  R  R  R  R  R  R  R  R  R  R  R  R  R  R | Younger  Younger  Younger  Younger  Younger  Younger  Younger  Younger  Younger  Younger  Elderly  Elderly  Elderly  Elderly  Elderly  Elderly  Elderly  Elderly  Elderly  Elderly |

**^a^**Sex: f = female; m = male

**^b^**Handedness: R= Right
